# A Gyroless Algorithm with Multi-Hypothesis Initialization for Projectile Navigation

**DOI:** 10.3390/s21227487

**Published:** 2021-11-11

**Authors:** Nabil Jardak, Ronan Adam, Sébastien Changey

**Affiliations:** French-German Research Institute of Saint-Louis, 5 Rue du Général Casssagnou, 68300 Saint-Louis, France; ronan.adam@isl.eu (R.A.); sebastien.changey@isl.eu (S.C.)

**Keywords:** gyroless, initialization, magnetometer, navigation, observability, projectiles

## Abstract

Projectiles are subjected to a high acceleration shock at launch (20,000 g and higher) and can spin very fast. Thus, the components of onboard navigation units must therefore withstand such constraints in addition to being inexpensive. This makes only a few inertial sensors suitable for projectiles navigation. Particularly, rate gyroscopes which are gun-hardened and have an appropriate operating range are not widely available. On the other hand, magneto-resistive sensors are inexpensive and can satisfy both gun-hardening and operating range requirements, making them an alternative for angular estimation in guided projectiles. This paper presents a gyroless navigation algorithm for projectiles. The lack of gyroscope is handled by the usage of attitude kinematics computed over past attitude estimates of the filter, coupled with a measurement model based on magnetometer and GPS observations of the attitude. The observability of the attitude when considering non-calibrated magnetometers and its dependency on the initialization is addressed. Then, to cope with the initialization dependency of the filter, we proposed a multi-hypothesis initialization algorithm. In terms of performance, the algorithm is shown to provide a high-rate navigation solution with an interesting performance.

## 1. Introduction

Multi-sensor data fusion has become the basis for precise navigation for consumer-grade navigation units based on inertial sensors. The first inertial sensors were mounted on a gimbal system that maintains the accelerometer fixed in the inertial frame. This system is heavy and costly and was replaced in mass-market applications by cost-, weight- and size-effective strap-down systems in which the IMU (Inertial Measurement Unit) is fixed to the platform. The inertial frame is then maintained analytically by tracking the platform attitude by means of gyroscope outputs integration. Such gyroscopes have to have a stable scale factor and the appropriate operating range and bandwidth for the application. To reach superior performance, navigation units based on consumer-grade IMUs are updated by exterior systems, such as, depending on the application, Doppler radar, seeker, star tracker and most recently in the last two decades by GPS.

Data fusion algorithms have largely been addressed in the literature (in [1], a background for attitude estimation solutions is provided). They aim generally at the determination of the position, velocity and attitude. While for some applications the attitude is a by-product of the coupling algorithm that allows us to maintain an inertial frame analytically, for others it is essential information. In space, the control of a spacecraft orientation is needed to be maintained in an appropriate direction [2,3]. Then, aerial and land applications have boosted the usage of multi-sensor navigation systems following the advent of MEMS sensors and the increase in computation capability and speed of embedded processors. For many years, coupled solutions have been extensively used in drones, and they have started to enter the rail market. Navigation is also needed for missiles and on-board guided projectiles in which the attitude and rotation rate information are required for stability and guidance [4].

Sensor selection for projectiles will depend on their quality (noise, bias repeatability, and misalignment), cost, weight, size, operating dynamic range, and also on their reliability and availability. In particular, sensors have to withstand acceleration at launch of many thousands of Gs (typically 20,000 Gs for a 155 mm projectile [5]) and fit within the projectile dimensions left for the guidance navigation and control (GNC) system. Those considerations mean only a few sensors are suitable for projectiles, and it is interesting to study navigation with a reduced configuration of sensors. Namely, a gyroless solution can be particularly interesting for projectiles as gun-hardened gyroscopes with a sufficient operating range are not commonly available. Moreover, the main angular rate for a projectile is at the roll component (contrarily to automotive where it is mainly at the yaw component), and its linear velocity is very high (typically Mach 1 to Mach 3). This will require a high-rate prediction step, typically several hundreds of Hz for aero-stabilized projectiles. 

On the other hand, it has been proven that magnetometers based on magneto-resistive technology can satisfy both gun-hardening and operating range requirements [6,7]. They are also inexpensive, which makes them a possible alternative for attitude estimation in guided projectiles. However, the Earth’s magnetic field measured by a magnetometer is distorted by the intrinsic errors of the sensor (bias, scale factor) but also by other perturbations due to the ferromagnetic material composing the projectile and the induced current due to the rotation of the metallic projectile [6,8]. As a result, the attitude (which is the orientation between the Earth’s magnetic field and the field measured by the 3-axis magnetometer aligned with the body frame) estimation based on magnetometer outputs will be affected by such distortions. In this paper, we assume that the errors in the magnetometer measurement include a multiplicative part (scale), an additive part (bias) and Gaussian white noise. 

The navigation filter state is usually augmented by sensors errors to prevent their propagation into the useful state components. However, a poor observation model can lead to filter divergence if not correctly taken into account. Usually, sensors errors are calibrated, and residuals are assumed to be small enough and are eventually removed from the state. This is plausible for many applications where errors stay almost constant between the calibration stage and the operational stage, but for projectiles the shock at launch makes sensors errors likely to change from their values obtained before the launch [5,8]. Thus, the beforehand calibration shall be completed with in-flight estimation of the sensor errors and especially for the magnetometers which are used for the attitude angles observation.

Several techniques have been proposed for online magnetometer calibration. Attitude-independent calibration methods [9,10,11,12,13] have been largely employed and are based on the assumption that the magnitude of the measured Earth’s magnetic field is constant regardless of the direction of the magnetometer. However, a proper spatial distribution of the magnetometer data is needed, and the calibration parameters are estimated in the sensor frame, as a rotational ambiguity exists in the solution. This issue can be solved by simplifying the magnetometer model, i.e., by neglecting the sensor misalignment with the carrier [13] or by assuming symmetric properties of the magnetic distortions [9,11]. This assumption strongly depends on the position of the magnetometer in the carrier and cannot always be satisfied. Some techniques try to solve this rotational ambiguity by the use of additional sensors: accelerometers by measuring the gravity field [14] or rate gyros [15].

Gyroless techniques for attitude estimation have been the focus of many papers. Sensors such as magnetometers and GNSS receivers have been used to observe the attitude or one of its components (the roll angle or pitch and yaw angles). In [16], the authors used a simplified 6DOF flight dynamic model of the projectile, updated with three-axis magnetometer and two-axis accelerometer sensors. The radial components of the magnetometer are used to observe the roll angle and determine the spin rate, while the axial magnetometer is used to observe the pitch angle. Radial magnetometer data have also been considered in [17] to derive pitch and yaw estimates based on the zero-crossing method and dynamic constraint equations. In [18], an attitude kinematic model using the body angular momentum of a spacecraft updated by a star tracker is developed. In [19] roll estimation is carried out using the GNSS signal power received by a side-mounted patch antenna. In [20], the attitude of a turntable is estimated by the demodulation of periodic oscillations of GNSS carrier phase measurements. Another technique [21], based on GNSS carrier phase measurement, implements differential positioning involving three antennas quite far away from each other. In [22], the attitude is obtained from a gyro-free INS made by six accelerometers arranged in a predetermined configuration around the center of mass. IMU processing for projectiles based on a set of six arranged accelerometers is also found in [23] which are used in conjunction with a three-axis magnetometer and hybridized with GNSS. 

In this paper, we study a gyroless algorithm for projectile navigation and specifically for large-caliber projectiles. To cope with the absence of a gyroscope, we reconstruct the angular velocity based on the derivatives of the attitude angles computed on the past attitude estimates. Then, the attitude is updated by the GPS-based pitch and yaw as well as the magnetometer observation of the attitude. Firstly, we carried out a preliminary analysis of the model in which the filter convergence dependency to the initialization was highlighted. This was followed by two solving methods. The first method consisted of inflating the filter covariance matrices to widen the initial domain of the state for which the state estimation errors stay bounded. The second method explored a multi-hypothesis initialization algorithm that avoids the drawbacks of the first method. We will use both simulations and the collected data to illustrate the algorithm behaviors and to analyze the results.

Consequently, the paper is organized as follows. A gyroless algorithm is presented in Section 2. Section 3 carries out a preliminary analysis of the algorithm in which the initialization dependency is highlighted, and then multi-hypothesis initialization is presented. Section 4 presents the performance predicted by the simulation. Section 5 presents the results obtained by using experimental data. Section 6 summarizes the main results.

## 2. Gyroless Navigation Filter

**Figure 1:**
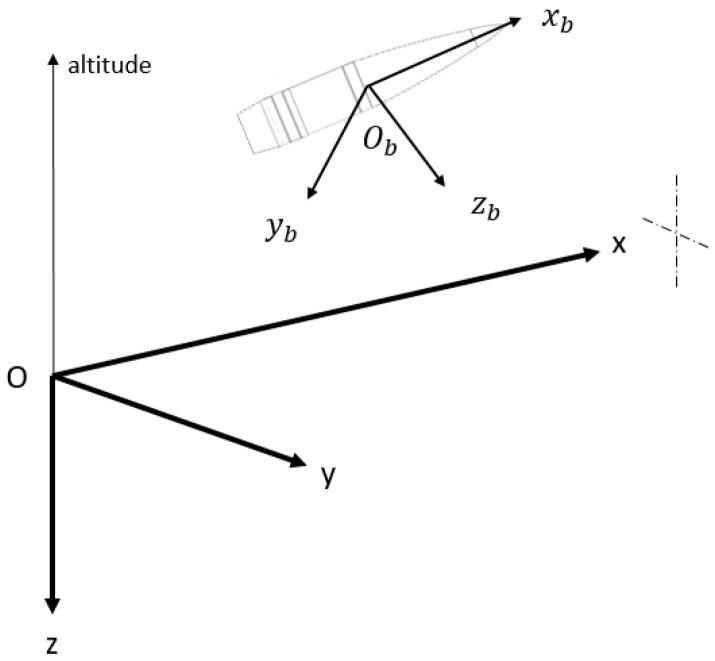
The local frame (*Oxyz*) and the body frame ($O_{b}$, $x_{b}$, $y_{b}$, $\;z_{b}$).

The model assumes a local navigation frame in which the position and the velocity will be expressed, and the attitude angles are also defined as the angles that allow the transformation between the body frame and the local frame. The local frame (*O*, *x*, *y*, *z*) is defined such that the XY Plan is tangential to the Earth’s ellipsoid at the launch position *O*, with (*Ox*) being aimed towards the target, (*Oz*) is vertical downward and (*Oy*) completes the direct coordinate system. The body frame ($O_{b}$, $x_{b}$, $y_{b}$, $z_{b}$) is centred on the projectile centre of mass, $(O_{b}x_{b}$) is the projectile longitudinal axis oriented toward the nose, the $Y_{b}Z_{b}\;$ plan is perpendicular to $(O_{b}x_{b}$). Both frames are visible in Figure 1. The three-axis accelerometer and three-axis magnetometer are assumed to be placed along the body frame axes. The filter used to integrate the sensors measurements is the usual Kalman Filter (KF) for which the error dynamics and the observation models will be expressed in this section.

### 2.1. Dynamic Model

The integrated navigation solution implements a KF based on the use of GPS, three-axis accelerometer and three-axis magnetometer sensors. A loose GPS/INS coupling is considered in which the magnetometer data are used in the observation model in addition to GPS position, velocity and GPS pitch and yaw. The state vector is defined as follows

(1) $$\delta x={\left[\delta p,\delta v,\delta \rho ,\delta b_{a},\delta b_{B},\;\delta S_{B}\right]}^{T}$$

where p is the 3D position and v the 3D velocity, both expressed in the local frame.$\;\rho ={\left[\phi \;\theta \;\psi \right]}^{T}$ is the attitude defined by the Euler angles (roll, pitch and yaw). $b_{a}$ is the accelerometer bias 3×1 vector, $b_{B}$ is the magnetometer bias 3×1 vector and $S_{B}$ is the magnetometer scale factor 3×1 vector, and all are expressed in the body frame. In-flight estimation of the sensor error reduces some in-factory calibration especially needed for projectile, where the calibration carried out before launch can vary following the gun acceleration shock at launch and/or due to an additional perturbation that could arise during the flight.

The spin rate of projectiles can be high. Usually, if a gyroscope is available, it is used to propagate the attitude between two updates by the GPS solution. Without a gyroscope, the attitude propagation will be performed using an approximate value of the angular velocity vector, computed on previous estimates of the attitude:

(2) $${\left(\omega_{bi}^{b}\right)}_{k}\approx {\left(\omega_{bi}^{b}\right)}_{k-1}+\Delta \omega$$

where $\omega_{bi}^{b}$ is the angular velocity vector computed by using the kinematics relating the body angular rates to the time derivatives of Euler angles as:

(3) $$\omega_{bi}^{b}=\left(\begin{array}{c} \dot{\phi }-\dot{\psi }\sin\theta \\ \dot{\theta }\cos\phi +\dot{\psi }\sin\phi \cos\theta \;\\ \dot{-\theta }\sin\phi +\dot{\psi }\cos\phi \cos\theta \end{array}\right)$$

In (3), the Euler angle derivatives are computed based on two past attitude estimates: ${\dot{\rho }}_{k}={\left[{\dot{\phi }}_{k}\;{\dot{\theta }}_{k}\;{\dot{\psi }}_{k}\right]}^{T}={\left(nT_{s}\right)}^{-1}\left(\rho_{k-1}^{\;}-\rho_{k-1-n}\right)$. $T_{s}$ is the time step of the accelerometer and magnetometer sensors, which is also the Kalman filter prediction time step. The time interval of the Euler angle derivatives calculation $nT_{s}$ must be small for the attitude velocity to stay accurate, but large enough to reduce the noise on it. Its value is thus a tradeoff between accuracy and precision. Δω is the angular velocity vector variation between successive values of $\omega_{bi}^{b}$. It is the error made by using previous estimates of the attitude in computing the current angular velocity vector. Its value is proportional to $T_{s}$.

The Euler angle derivatives are calculated with a delay, and the relative error is neither white nor stationary because it depends on the angular dynamics of the projectile. Taking into account this deviation from the Kalman filtering assumptions should improve the filter performance, but in the following state error model, this term will be considered as Gaussian white noise $w_{\rho }$. The resulting cumulative attitude prediction error will be corrected by the regular update step of the filter. At the start of the fusion algorithm, the attitude velocity is initialized with an a priori knowledge of the roll velocity at the muzzle exit, which depends on the type and number of propelling charges. The pitch and yaw derivatives are well approached by zero: ${\dot{\hat{\rho }}}_{1}={\left[{\dot{\phi }}_{0}\;,\;0,0\right]}^{T}$. The system dynamics are written as [24]:

(4) $$\begin{array}{l} \delta \dot{\rho }=w_{\rho }\\ \delta \dot{p}=\delta v\\ \delta \dot{v}=-\left({\hat{R}}_{b}^{l}{\tilde{f}}^{b}\times \right)\delta \rho +{\hat{R}}_{b}^{l}\delta b_{a}+{\hat{R}}_{b}^{l}\eta_{a}\;\\ \delta \dot{b_{a}}=-\beta_{a}\delta b_{a}+w_{a}\\ \delta \dot{b_{B}}=-\beta_{B}\delta b_{B}+w_{B}\\ \delta \dot{S_{B}}=-\beta_{S}\delta S_{B}+w_{S}\; \end{array}$$

The accelerometer and magnetometer biases and scale factors are commonly modelled as a first-order Gauss–Markov process where $w_{a},w_{B},\;w_{S}$ are centered white Gaussian processes and $\beta_{a}$, $\beta_{B},\;\beta_{S}$ the inverse of the respective correlation times of sensors errors. $\eta_{a},\eta_{B}$ are the accelerometer and magnetometer noises modelled as centred white Gaussian processes. ${\tilde{f}}^{b}$ is the specific force measured by the accelerometer (m/s²), it is corrected for the accelerometer bias ${\hat{b}}_{a}$ estimated by the algorithm $f^{b}={\tilde{f}}^{b}-{\hat{b}}_{a}$. ${\hat{R}}_{b}^{l}$ is the direct cosine matrix accounting for the orientation between the body frame and the local frame. (.×) is the skew-symmetric matrix operator. The acceleration in the local frame is obtained by adding the acceleration due to gravity $g^{l}$: $a^{l}={\hat{R}}_{b}^{l}f^{b}+{\left[0\;0\;g^{l}\right]}^{T}$ (for short flights the Coriolis term and the orientation change of the local frame w.r.t. the Earth are neglected). The position $p^{l}$ in the local frame is obtained by integrating the velocity $v^{l}$, itself calculated by the integration of $a^{l}$. The analysis of the dynamics shows that an initial attitude derivative error causes an attitude divergence, while an initial attitude error and accelerometer biases cause an error in the velocity. Without aiding, the solution diverges with time and the system’s nonlinearity increases. In the Kalman filter, the aid is performed by using accurate measurements. If, in addition, the state is observable, the state error is kept small and the dynamic linearization should be satisfied. High linear and angular dynamics of projectiles require the use of a small integration time to reduce system integration errors. At the beginning, the initial uncertainties dominate the error. As time goes on, the integrated noise becomes dominant and, without state updates, the error will grow more or less quickly depending upon the accelerometer quality and the a priori knowledge of the attitude velocity. The next paragraph defines the observation model of the filter.

### 2.2. Observation Model

Two observation models are considered related to the fact that magnetometer measurements are available at a higher rate (typically > 100 Hz) than GPS position solution (typically ≤ 10 Hz). First, at each reception time of magnetometer measurements outside the epochs of GPS measurements, the measurements are given by the difference between the Earth’s magnetic field in the local navigation frame $B_{E}^{l}$, which can be obtained from the World Magnetic Model (WMM) [25], and the magnetometers outputs ${\tilde{B}}^{b}$ corrected by the magnetometer bias ${\hat{b}}_{B}$ and scale factor ${\hat{S}}_{B}$ estimates and converted into the local frame as $\delta B^{l}=B_{E}^{l}-{\hat{R}}_{b}^{l}{\left(I_{3}+{\hat{S}}_{B}\right)}^{-1}\left({\tilde{B}}^{b}-{\hat{b}}_{B}\right)$. By using ${\hat{R}}_{b}^{l}=\left(I_{3}-\left(\delta \rho \times \right)\right)R_{b}^{l}$ and writing the magnetometer measurement as ${\tilde{B}}^{b}=\left(I_{3}+S_{B}\right)B^{b}+b_{B}+\eta_{B},$ it happens that:

(5) $$\delta B^{l}=\left(-{\hat{B}}^{l}\times \right)\delta \rho -{\hat{R}}_{b}^{l}{\left(I_{3}+{\hat{S}}_{B}\right)}^{-1}\left(\delta S_{B}B^{b}+\delta b_{B}+\eta_{B}\right)$$

Second, at the instants of GPS output availability, the observation model is formed, in addition to (5), by the GPS position, velocity and the pitch and yaw derived from the GPS velocity vector:

(6) $$\begin{array}{c} \delta p^{l}=p_{INS}-{\tilde{p}}_{GPS}+\eta_{p}\\ \delta v^{l}=v_{INS}-{\tilde{v}}_{GPS}+\eta_{v}\\ \delta \theta =\theta_{INS}-{\tilde{\theta }}_{GPS}+\eta_{\theta }\\ \delta \psi =\psi_{INS}-{\tilde{\psi }}_{GPS}+\eta_{\psi } \end{array}$$

where the INS subscript corresponds to the last prediction of the state and η denotes the measurement noise. Assuming that the velocity vector is parallel to the projectile main axis (i.e., small angle of attack), the GPS pitch and yaw can be derived from the GPS velocity in the local frame according to:

(7) $$-\frac{\pi }{2}\leq {\tilde{\theta }}_{GPS}=\tan^{-1}\left(\frac{-{\tilde{v}}_{z}^{l}{}_{GPS}}{\sqrt{{\tilde{v^{l}}}_{x}{{}_{GPS}}^{2}+{\tilde{v^{l}}}_{y}{{}_{GPS}}^{2}}}\right)\leq \frac{\pi }{2}$$

(8) $$-\pi \leq {\tilde{\psi }}_{GPS}=\tan^{-1}\left(\frac{{\tilde{v}}_{y}^{l}{}_{GPS}}{{\tilde{v}}_{x}^{l}{}_{GPS}}\right)\leq \pi$$

Combining GPS pitch and yaw and magnetometer measurements in the observation model allows us to observe the attitude which should relax the constraint of the attitude initialization. To assess the covariance between the GPS velocity and the GPS pitch and yaw, we carried out numerical simulations using the projectile trajectory of Section 3.1 and by assuming a normal distribution of the GPS velocity error. We have found that the covariance between GPS pitch and yaw noises and GPS velocity noise are more than two orders of magnitude smaller than the velocity noise variance. The measurement covariance matrix ***R*** can then be assumed as diagonal:

(9) $$R=cov\left(\eta_{p},\;\eta_{v},\eta_{\theta },\;\eta_{\psi },\;\eta_{B}\right)=diag\left(\sigma_{p}^{2}\;,\;\sigma_{v}^{2},\;\sigma_{\theta }^{2},\;\sigma_{\psi }^{2},\;\sigma_{B}^{2}\right)$$

This observation model differs from existing gyroless GPS-based algorithms [26] by the only magnetometer-based update step between two GPS data epochs and by the non-use of the accelerometer in the observation model, which was replaced by the GPS pitch and yaw. The first ensures a high update rate of the attitude along with magnetometers errors, while the second avoids managing the correlation between the system noise process and the measurement noise process.

## 3. Filter Preliminary Analysis

### 3.1. Scenario Description

In order to assess the performance, we generated synthetic sensor data based on representative simulation tools. The sensor data are modelled using truth trajectory model and error models. The estimated Position Velocity Attitude (PVA) solution and truth data are the input for a performance assessment tool which derives PVA performance statistics of the gyroless algorithm.

#### 3.1.1. Truth Trajectory

**Figure 2:**
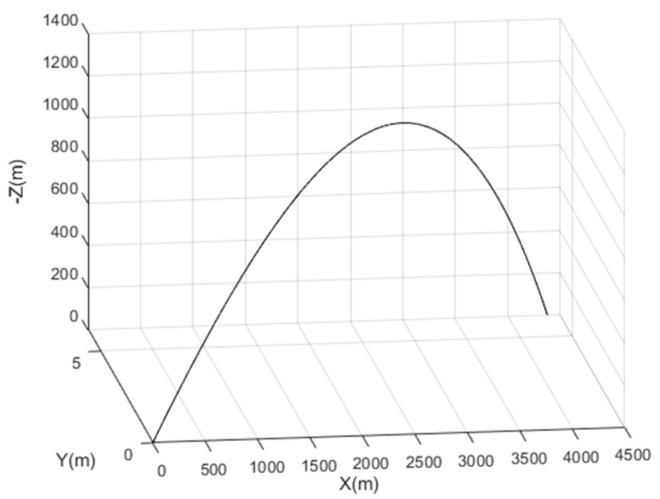
Trajectory in the local frame.

The projectile trajectory was generated based on a vehicle dynamic model, Balco. This is a six- and seven-degree-of-freedom (6-7DOF) trajectory simulation program based on the mathematical model defined by the NATO Standardization Recommendation 4618 [27]. The primary goal of Balco is to compute high-fidelity trajectories for both conventional and precision-guided projectiles. The outputs of Balco software tool used in this work are the position, the velocity, the Euler attitude angles, the actual accelerometer and magnetometer outputs, as well as the angular velocity vector (this latter will be used only for performance comparison). Figure 2 illustrates the simulated free flight trajectory with the local frame defined such that (Ox) is 120° from the north direction clockwise in the Earth’s tangent plane and (Oz) is vertical downward.

#### 3.1.2. Sensor Data

The 1 kHz accelerometer and magnetometer data at the output of the Balco software tool were perturbed by the following error model [28]:

(10) $$\tilde{y}=\left(I_{3}+M_{3}\right)\cdot y+b_{y}+n_{y}\;$$

where y is the 3×1 true sensor output in the body frame, $M_{3}$ is a 3 × 3 matrix that represents the misalignment error (off-diagonal terms) and sensitivity error (diagonal terms), $b_{y}$ is the 3 × 1 sensor bias vector and $n_{y}$ is the 3 × 1 sensor noise vector (assumed zero mean Gaussian). Table 1 defines the values of the model parameters. These correspond to residuals after calibration (see Section 5 for the calibration method). Later, we will add biases on magnetometer scale factors and biases on top of the residuals to study the observability of the filter.

#### 3.1.3. GPS Data

A configuration consisting of a low-cost GPS L1C/A receiver providing data with a 10 Hz rate was adopted. A software GPS simulator tool was used in order to generate the PVT (position, velocity and timing) based on the true trajectory generated by the Balco software tool and a satellite orbit description file. The obtained position and velocity performances of the loose coupling scheme with the GPS PVT solution are compliant with the expected performance of a standalone single-frequency/single-constellation GPS-L1C/A positioning and they will not be presented. Rather, we will focus the analysis on attitude estimation.

### 3.2. Initialization Effect on the Filter Convergence

We study the feasibility of magnetometers errors estimation in flight by means of the filter defined in the previous section, which uses non-calibrated magnetometer data. The filter state includes both magnetometer bias and scale factor error vectors which have been set to S = [0.2, 0.15, −0.25] and b = [0.2, 0.06, −0.05] Gauss, respectively, in the output model of the magnetometer. They are added on top of the residuals given in Table 1, making the values of the scale factor S and the bias b composed of a constant component and a random component. 

**Figure 3:**
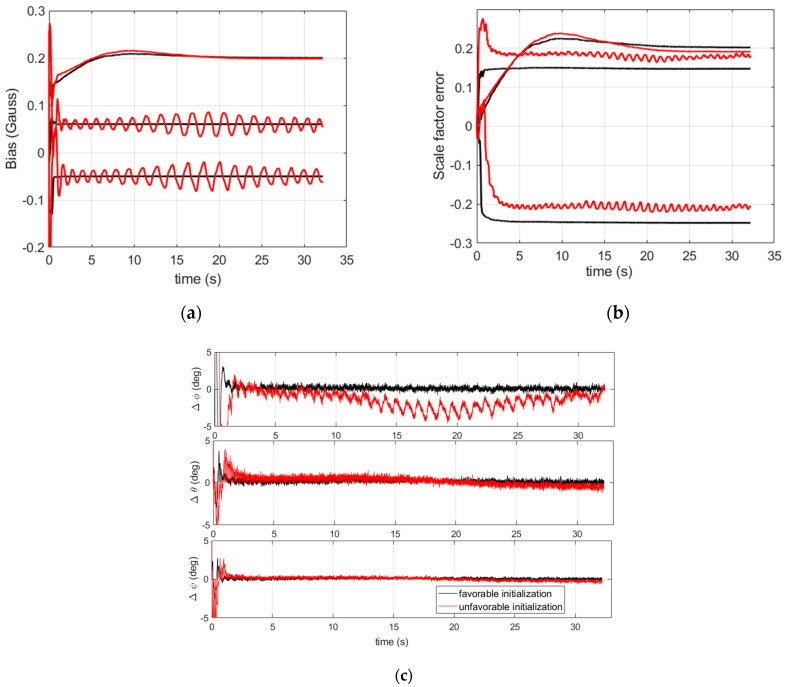
Comparison between favorable and unfavorable attitude initialization. (**a**) Magnetometer bias estimates; (**b**) magnetometer scale factor error estimates; (**c**) attitude errors. Case of a favorable attitude initialization combined with small covariance matrices (black curves) along with unfavorable attitude initialization combined with inflated covariance matrices (red curves).

We recall that the filter defined in the previous section is a linear time varying system in which the magnetometer errors and the attitude are coupled in the magnetometer measurement model. The observability of these states will be studied simply by simulation in the two cases of a favorable and an unfavorable initial condition. Assume that an approximate initial value of the attitude is available (the favorable case). This should allow for an easier identification of the scale factors, biases and the attitude by the algorithm. Therefore, we can use optimal (small) process noise and measurement covariance matrices Q and R, and we obtain relevant estimations for the attitude and magnetometer errors, as shown in Figure 3 (black curves). When considering an unfavorable initial value of the attitude, the weak observability of the system in terms of attitude and magnetometer error separation requires us to sufficiently increase the covariance matrices. Indeed, increasing the covariance matrices will enlarge the initial domain for which the state estimation errors stay bounded in favor of filter stability [29]. In this case, we found that the filter stability is obtained whatever the initialization condition, but this comes at the expense of a degraded attitude performance, as is shown by the comparison in Figure 3 (red curves) where the degradation is pronounced for the roll angle. Henceforth, the scale factor error is estimated inaccurately, which leads to oscillating radial magnetometer errors and inaccurate roll estimations. Notice that such a distortion is actually dependent upon how big the covariance matrices are inflated. In practice, during the filter validation step, the covariance matrices can be increased gradually until achieving a convergence with an acceptable distortion for the application.

### 3.3. Multi-Hypothesis Initialization Estimator

**Figure 4:**
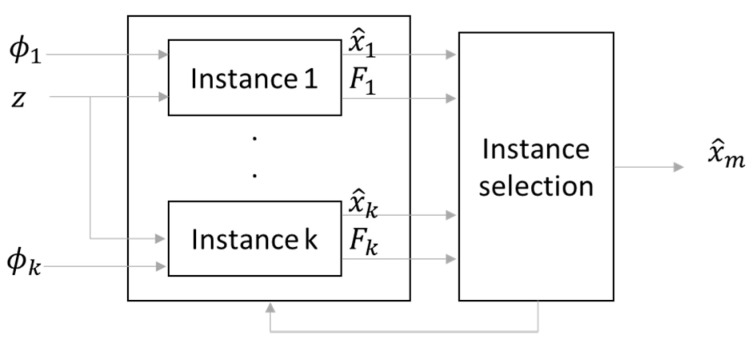
Principle of the multi-hypothesis initialization estimator.

In order to cope with the initialization dependency of the filter while avoiding a significant covariance inflation, multiple instances of the KF can be run in parallel over a short time following the start time of the mission (see Figure 4). Each instance is initialized with a different starting value of the roll component ($\phi_{k}$ for the *k*th instance), receives the measurement input (z) and produces an estimate of the state (${\hat{x}}_{k}$) as well as a convergence indicator computed on measurement residuals ($F_{k}$). Instances of the filter with bad initial values of the roll angle will have larger indicator values than the instances with favorable initial roll values. Hence, a decision function identifies the instance with the best convergence indicator, which will be maintained until the end of the mission and, at the same time, kills all the other instances for the rest of the mission. The final state is the one obtained at the output of the winning instance. The convergence indicator of the KF is set to be the sum of the squared residuals (SSR) of a single component of the Earth’s magnetic field in the local frame, normalized by the residual magnetometer measurement variance $\sigma_{m}^{2}$. This value is taken from the residual covariance matrix $HPH^{T}+R$ for a filter having converged (correctly initialized); thus, it is determined beforehand during the filter tuning step and provided to the filter bank. Note that the convergence detection is based on the magnetometer measurement residual because any bad attitude estimate will shift the expected magnetic field w.r.t. the measured field. The convergence indicator is computed over an interval of n samples taken once the filter is supposed to have converged ($i\geq \;n_{o}$):

(11) $$F=\;\sum_{i=n_{o}}^{n_{o}-1+n}\frac{{\left(\delta B_{i}^{l}\right)}^{2}}{\sigma_{m}^{2}}\;$$

The filter convergence criterion is reached when the indicator F is below a threshold $F_{th}$, $F<F_{th}$. Actually, the filter that will be selected in the filter bank to run over the whole scenario is the one with the smallest F among the filters that passed the convergence test. $F_{th}$ can be designed as a function of a probability of false alarm, $p_{FA}$ as $F_{th}=f^{-1}\left(1-p_{FA}\right)$, where f is the cumulative density function of the Chi square distribution of *n* degrees of freedom.

**Figure 5:**
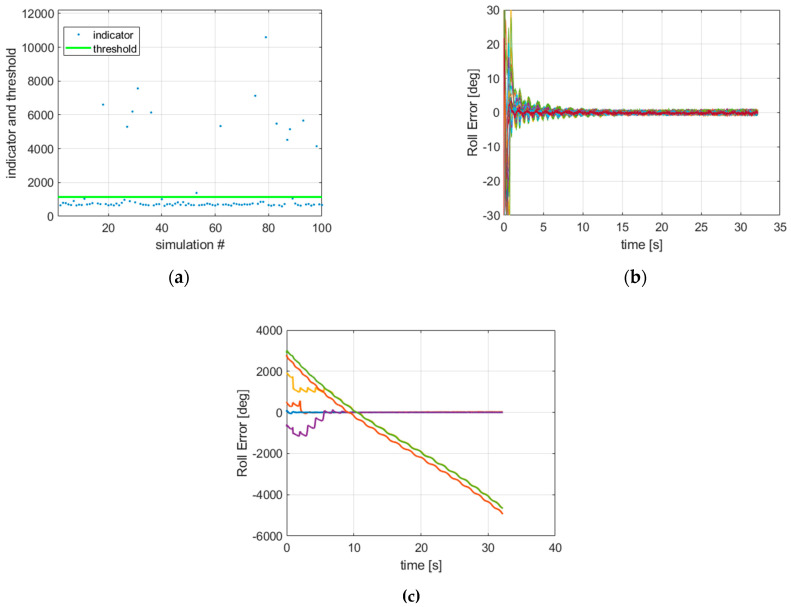
Results of a single filter initialized by random roll angle values. (**a**) Convergence indicator with convergence threshold; (**b**) roll estimation errors of the simulations that passed the test; (**c**) roll estimation errors of the filters that failed the test (note here the roll error has been unwrapped to allow the divergence to be seen graphically).

**Figure 6:**
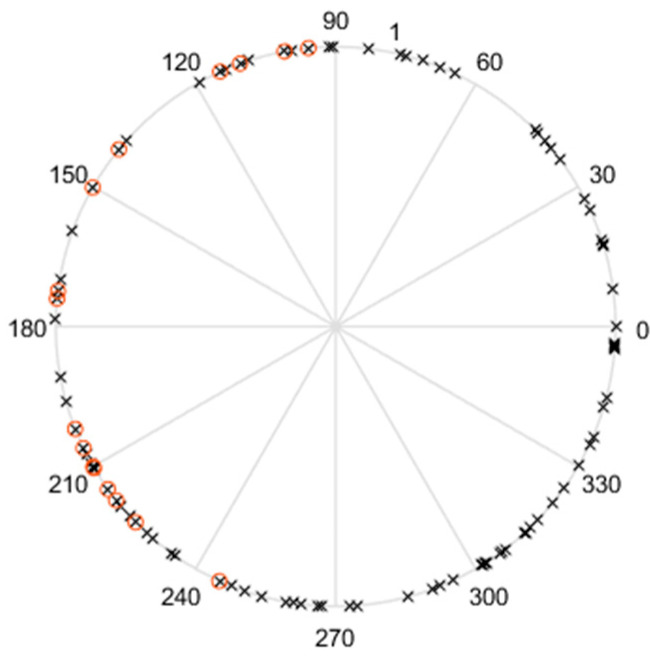
Initial roll angle errors (degrees) given by the crosses x. The values that failed the test are encircled.

In order to study the multi-hypothesis initialization filter bank, 100 simulations were run using firstly a single filter where the initial roll error was selected randomly in the interval [−180°, 180°]; the pitch and yaw angles errors were selected randomly in the interval [−15°, 15°]. This is because the initial pitch and yaw can be derived from the inclination and heading of the canon, and both are assumed to be known better than this uncertainty range. Based on the convergence criterion results, we plotted the roll error for the simulations which passed the convergence test and the ones which failed the test. Results are illustrated in Figure 5. We have checked that when the convergence indicator was below the threshold, the filter outputs converged well. Contrarily, the filter outputs diverged for the simulations where the indicator was above the threshold. Figure 6 shows the initial roll angle errors that were used and highlights the values that failed the test. These occur in the interval [90°, 270°] (but note that not all the values in this interval are bad initialization points). This suggests that running four filters initialized with 90° offset roll angles (for instance $\phi_{0}=0{}^{\circ},\;90{}^{\circ},\;180{}^{\circ},\;270{}^{\circ}$) should guarantee that at least one filter will pass the test.

**Figure 7:**
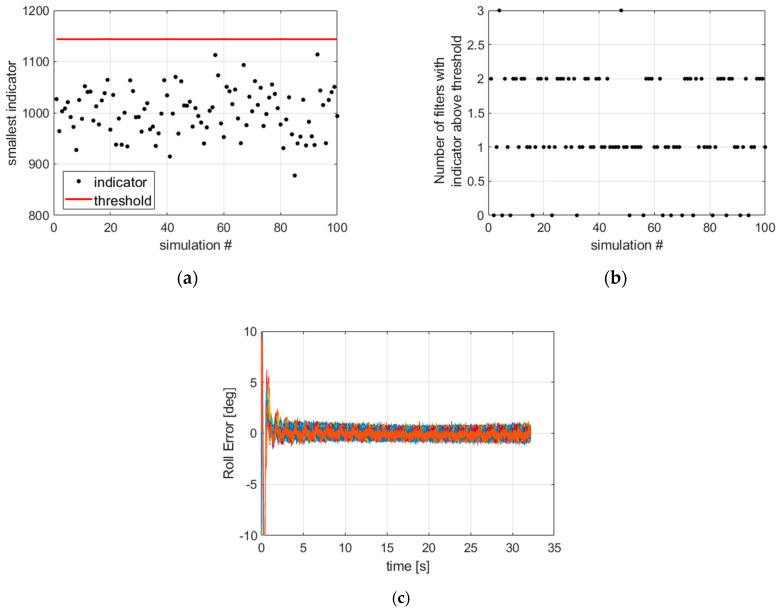
(**a**) The smallest indicator among the four filters is always below the threshold (corresponding to a pfa of 0.001); (**b**) the number of filters with indicator above threshold shows there is no case where the four filters failed at the same time; (**c**) 100 curves of the roll error at the output of the filter bank showing that all the simulations have converged.

We run 100 simulations using four filters in parallel, initialized respectively with a roll angle of 0°, 90°, 180°, 270°. The results are depicted in Figure 7. First, the smallest convergence indicator value among the four filters is shown to be below the threshold for all the simulations. Second, the number of filters with a divergent attitude estimate does not exceed three, suggesting that at each time there is at least one filter with a favorable initial attitude. The last image shows the roll error obtained at the output of the filter bank. We can verify the good convergence of all simulations (whatever the roll initialization error). The drawback of a multiple hypothesis initialization is the number of filters to be run in parallel at the beginning of the mission. In order to keep the computation load acceptable, the filter bank can be run using a reduced sampling frequency over the first few seconds. Once the filter with the smallest convergence indicator below the threshold is identified, all filters are switched off and the state and covariance of the identified filter are used to initialize a new filter operating at a more convenient sampling frequency for the remaining part of the flight.

## 4. Performance Assessment

In this section, we present the performance of the filter bank with multi-hypothesis initialization in terms of estimation of the attitude, of reconstructed angular velocity vectors and of magnetometer biases and scale factors. To this end, the same scenario described in the previous section was adopted. Namely, the magnetometers are supposed to be calibrated beforehand and the calibration residuals were simulated based on the 1-σ values of Table 1. Then, we added constant biases and scale factors on top of the residuals to simulate an additive distortion that could be created by the acceleration shock following the projectile launch. We assessed the performance of the filter based on 100 numerical simulations in which we modified the random components of the sensor data and the GPS start time of week. The filter bank is composed of four filters initialized with roll angles shifted by 90° as explained in the previous section with the first filter roll angle set randomly in the intervals [−180°, 180°]. The initial pitch and yaw angles were set randomly in [−15°, 15°], which are common to the four filters of the bank. We computed the attitude derivative in the filter over an interval of $nT_{s}$ = 0.5 s.

**Figure 8:**
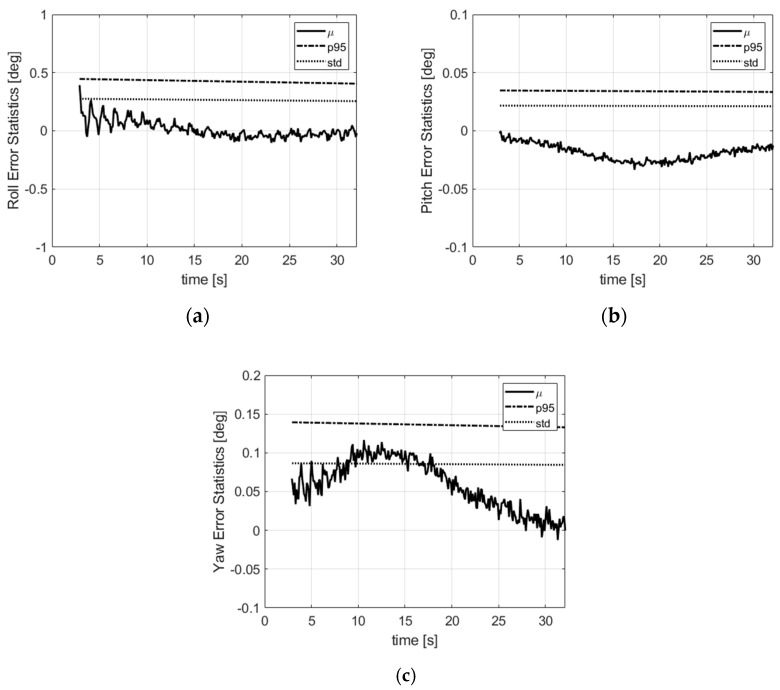
Attitude error statistics: (**a**) roll; (**b**) pitch; (**c**) yaw. µ is the mean error, std is the standard deviation, p95 is the 95% deviation w.r.t. the mean.

In Figure 8, we show the mean attitude error, the standard deviation (std) and the 95% offset with respect to the mean. The global error (mean value plus 95% offset) is lower than 1°, 0.1° and 0.2°, respectively, for the roll, pitch and yaw angles. This is thanks to the attitude propagation performed on the attitude derivative which is computed over past attitude estimates of the filter and the direct attitude observation in the measurement model. The mean error curves show small distortions which originate mainly from the scale factor and misalignment residual errors of the magnetometers that are not taken into account in the algorithm.

**Figure 9:**
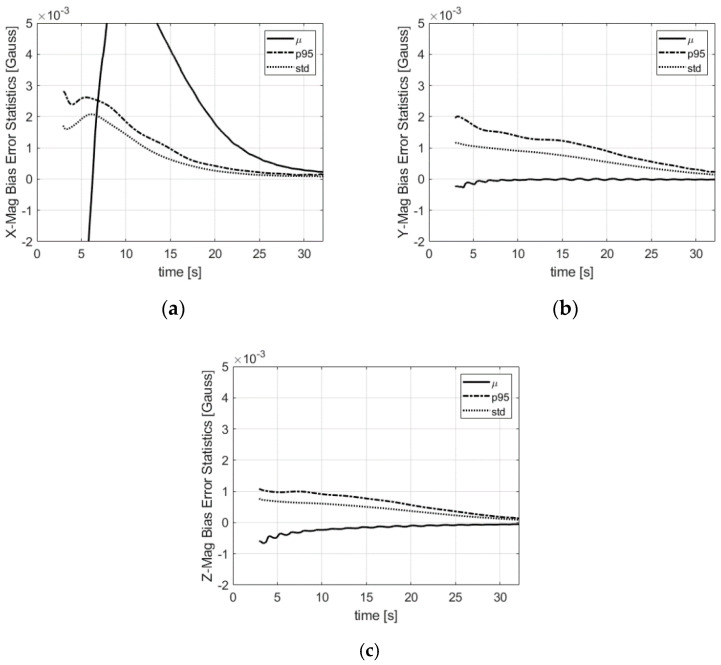
Magnetometer bias error statistics in the body frame: (**a**) x-direction; (**b**) y-direction; (**c**) z-direction. µ is the mean error, std is the standard deviation, p95 is the 95% deviation w.r.t. the mean.

**Figure 10:**
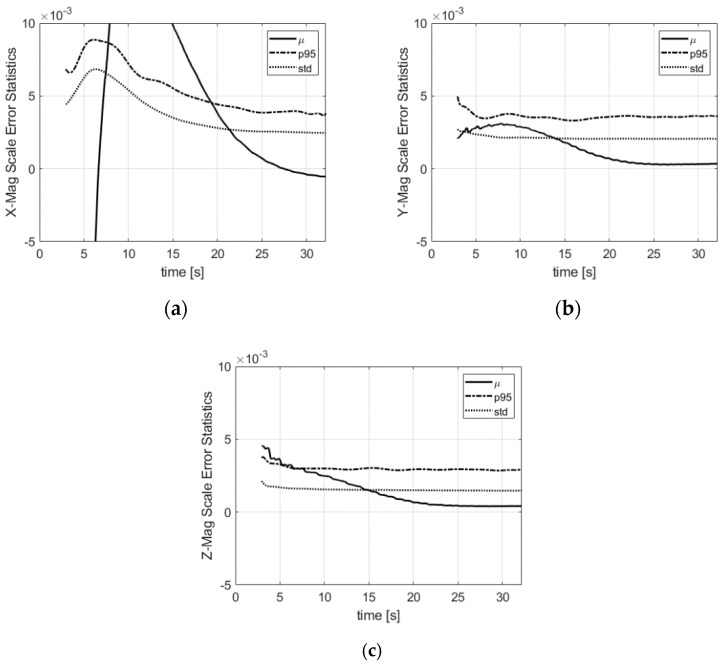
Magnetometer scale factor error statistics in the body frame: (**a**) x-direction; (**b**) y-direction; (**c**) z-direction. µ is the mean error, std is the standard deviation, p95 is the 95% deviation w.r.t. the mean.

The bias error statistics of the magnetometers are shown in Figure 9. The error spread after convergence is smaller than 1 milliGauss (1-σ) in the three directions. The oscillations of the radial biases are due to the scale and misalignment residuals combined with the roll dependence of the radial magnetometer outputs. The axial component of the magnetometer is pitch and yaw-dependent only. The magnetometer scale factors illustrated in Figure 10 are also estimated with a small error.

**Figure 11:**
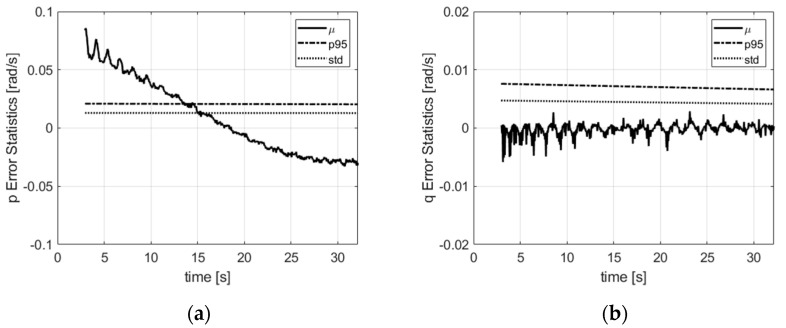

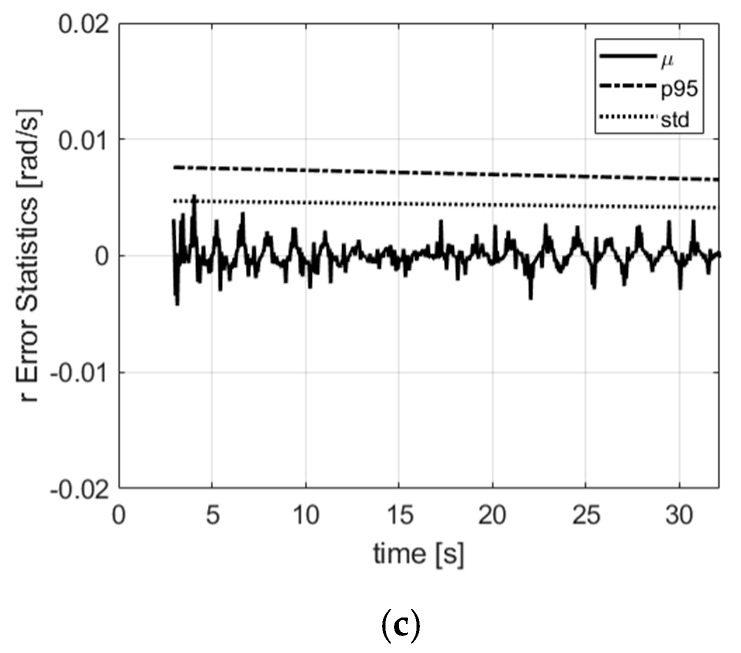
Body-frame angular velocity error statistics: (**a**) x-direction; (**b**) y-direction; (**c**) z-direction. µ is the mean error, std is the standard deviation, p95 is the 95% deviation w.r.t. the mean.

All the states that are dependent on the attitude are well estimated whatever the initial attitude thanks to the filter with multi-initialization coupled with the convergence detector, the direct observation of the attitude by the three-axis magnetometer and the GPS pitch and yaw. Not requiring an attitude initialization is important for projectiles because, while the initial pitch and yaw (which respectively represent the azimuth and inclination of the canon) could be known, the roll angle is not under control at the filter start time, which comes after the projectile leaves the canon. At this time, the projectile is in motion and the traditional accelerometer levelling does not apply. This stresses the essential role the magnetometer sensor can play in projectile navigation. Compared to the traditional GPS/INS algorithm [30] where the attitude is inferentially determined through the system dynamic, in this algorithm, the attitude converges rapidly as it is directly observed. The angular velocity vector is illustrated in Figure 11. The angular velocity error is below 0.05 rad/s along the main axis and is smaller than 0.01 rad/s in the radial directions. The curve shapes follow the angular dynamic shape on each axis, and are hence quite regular in the axial direction and sinusoidal in the radial directions. They are explained by the latency in computing the attitude derivative by the filter in the prediction.

## 5. Experimental Results

**Figure 12:**
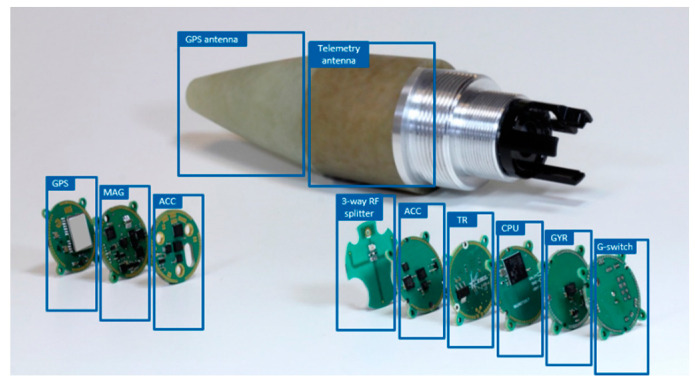
Projectile fuse and the instrumented electronics. In the figure: TR is the telemetry transceiver, G-switch is used to power-on the electronics following the launch. GPS antenna radome is located in the front part of the fuse followed by the telemetry antenna radome.

In this section, we test the algorithm with real data which were collected on board a projectile. The projectile was instrumented with consumer-grade sensors consisting of a three-axis anisotropic magnetoresistance (AMR) magnetometer, a three-axis MEMS accelerometer, a three-axis MEMS rate gyroscope and an L1-C/A GPS receiver. The sensor specifications are similar to those shown in Table 1. A photo of the projectile fuse along with the sensors and the different printed circuit boards (PCB) is provided in Figure 12.

**Figure 13:**
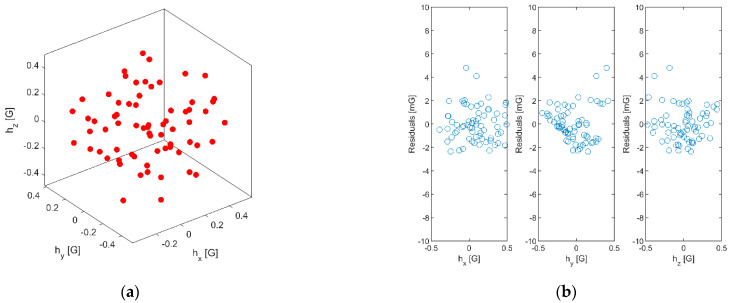
(**a**) Generated static magnetic fields; (**b**) calibration residuals.

The calibration of the magnetometers was performed using a 1.3 m diameter three-axis Helmholtz coil supplied by *Bartington Instruments.* The system also includes a close-loop controller and enables the mitigation of magnetic perturbations due to power lines or other electric devices as well as the cancellation of the Earth’s magnetic field. The device is used to generate predefined static magnetic fields during 800 ms for each different value. The magnetometer measurements are sampled at 8 kHz with 12-bit digital resolution, and a time average is computed for each generated magnetic field. The parameters of the magnetic field model are then estimated using a linear least squares optimization. Generated magnetic field and calibration residuals for one example can be seen in Figure 13.

In order to derive a reference for the attitude angles, we first post-processed the real data by an existing navigation program developed by ISL [16]. This program implements a 6DOF flight dynamic model which is updated based on a three-axis magnetometer and a three-axis accelerometer in order to estimate the attitude angles of a projectile. The attitude estimated by this program on real flight data have already been validated. This justifies the usage of this tool as a reference to assess the performance of the studied algorithm.

**Figure 14:**
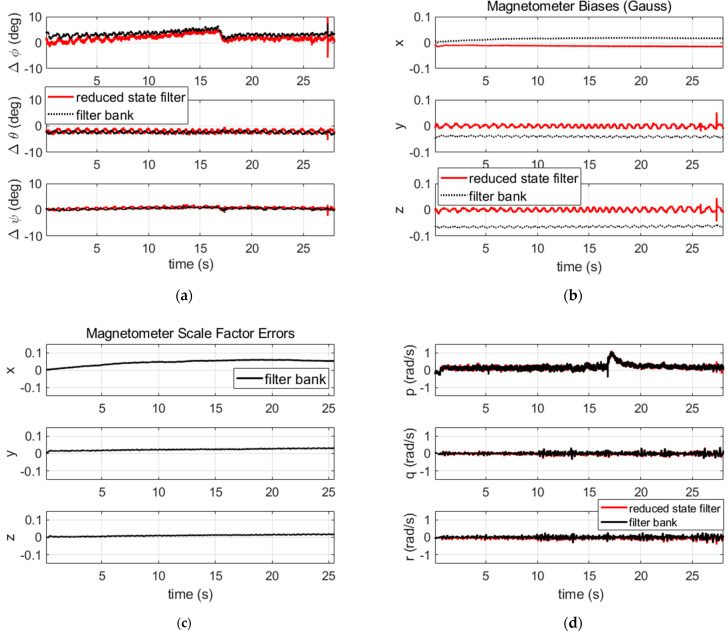
(**a**) Attitude errors; (**b**) magnetometer biases; (**c**) magnetometer scale factor shift; (**d**) angular velocity errors. Filter with a reduced state using calibrated magnetometer outputs (red curves) versus full state filter (number 2 in the bank) using non-calibrated magnetometer outputs (black curves).

We post-processed the sensor data first through the filter with a reduced state, which does not include the magnetometer scale factors in the state and which uses the calibrated magnetometer data. The results, plotted in Figure 14 (red curves), show that the estimated attitude is consistent with the attitude of the reference algorithm. The attitude error variation visible in the middle of the flight corresponds to a dynamic change of the projectile trajectory at that time. The mean value of the attitude difference is below 3° on roll, 2° on pitch and 1° on yaw. The pitch and yaw angles are observed in the filter by the magnetometer output and the GPS velocity-based pitch/yaw solution as well. Any imperfection in these measurements will lead to a biased estimation of the pitch/yaw angles and propagates into the roll angle estimate. Particularly, the GPS velocity is perturbed by the GPS antenna rotation, and the GPS data present a time misalignment of 10 ms with magnetometer data time (both were neglected in the simulated scenario of the previous section). We notice also that we used the raw sensor data without any “cleaning”. Such data usually shift from the theoretical model. Filtering this data cautiously for prior usage in the KF could bring some improvement. The estimated magnetometer biases are quite centered, which is a result of using calibrated magnetometer outputs. The oscillations observed in the radial directions during the simulations are confirmed. We compare the estimated angular rate [p,q,r] to the data recorded by an embedded gyroscope. The comparison illustrated on the same figure (the red curves, which are coincident with the black curves in Figure 14) shows the angular velocity components are correctly estimated.

**Figure 15:**
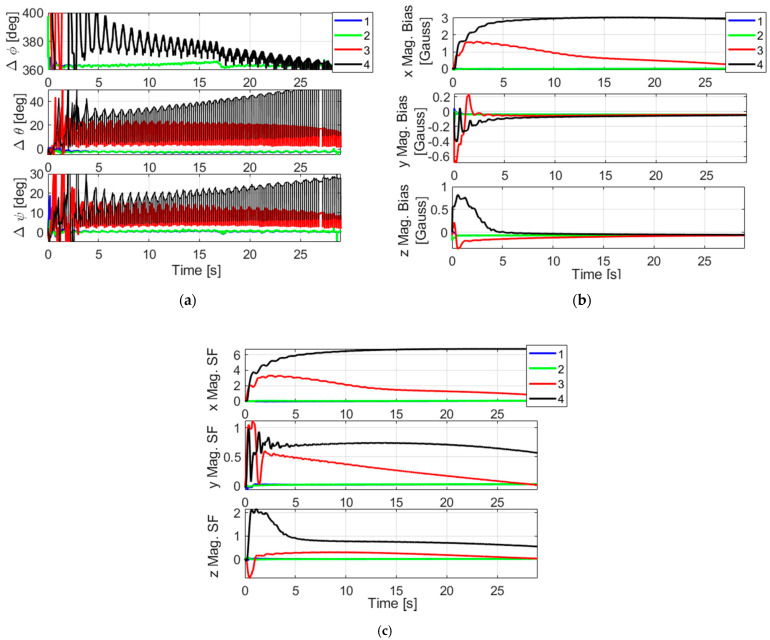
(**a**) Attitude error (difference with the reference algorithm); (**b**) magnetometer biases; (**c**) magnetometer scale factors. Results of the filter bank based on four filters. The green and the blue curves are superimposed after convergence.

We then provided the non-calibrated magnetometer data to the bank of four filters (here, the magnetometer scale factors are estimated by each filter). The roll angles of the filters are initialized with 0°, 90°, 180°, 270°. All filters were allowed to run until the end of the scenario for analysis purposes. The values of the convergence indicators we obtained are 1020, 877, 67,896 and 102,191, respectively, for the filters 1, 2, 3 and 4, respectively, while the threshold has a value of 1143 (relative to a pfa of 0.001). This suggests that filters 1 and 2 will pass the convergence test, but not filters 3 and 4. This is consistent with the results of Figure 15 where we can see the attitude error, the magnetometer biases and the scale factors estimated by each filter in the bank. The attitude error of filters 1 and 2 are quite similar except during the beginning of the scenario where they have different transients following different roll starting positions. Filters 3 and 4 led to large errors in attitude and magnetometer error estimations. For these filters the pitch and yaw estimates have large discrepancies, despite the updates by the GPS pitch and yaw that try to readjust them.

**Figure 16:**
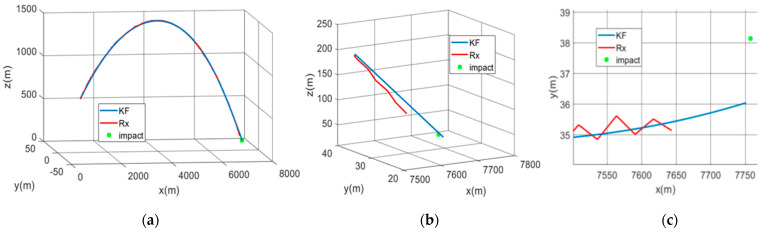
(**a**) The estimated 3D trajectory in the local frame (KF) along with the raw GPS receiver trajectory (Rx) and the actual position of impact; (**b**) zoom in of (**a**) around the end of the trajectory; (**c**) projection of (**b**) in the x-y plane.

The attitude results of filter 2 (which has the smallest convergence indicator) are illustrated in Figure 14 (black curves) and can be compared to the results of the unique filter with a reduced state and using calibrated magnetometer inputs. The attitude is seen to have a similar performance in both cases. We can also check in the same figure that the magnetometer biases are no longer centered in this case (use of non-calibrated magnetometer data). In terms of the positioning result, the trajectory estimated by filter 2 is shown in Figure 16 along with the raw GPS solution and the actual position of the impact. Here, in order to cope with the lack of sensor data during the last ~0.4 s of the flight, the estimated trajectory was extrapolated down to the height of the actual impact point. This results in an estimated impact position that is 6 m (2D) from the actual impact position.

Overall, the experiments validated the expectations and strengthened the conclusions drawn in this article. Principally, the benefit of the multi-hypothesis initialization filter bank for weak observability systems is highlighted, as well as the benefit of using magnetometers aside from GPS for high-rate attitude estimation when a gyroscope is not available.

## 6. Conclusions

In this paper we have presented a gyroless navigation algorithm for projectiles based on a low-cost GPS receiver and consumer-grade accelerometers and magnetometers. The algorithm performs an attitude prediction based on the attitude kinematics computed over past attitude estimates of the filter. This is possible since the attitude is observed by magnetometer measurements and GPS velocity-based pitch and yaw. The attitude prediction is necessary for tracking the spin rate of projectiles and to provide high-rate filter outputs. We have shown that the filter that estimates the magnetometer biases and scale factors in addition to the attitude requires the use of inflated KF covariance matrices in the case where a good initial attitude value is not available. This inflation allows the filter to converge but leads to a sub-optimal attitude estimation. In order to cope with the initialization dependency of the filter while avoiding covariance inflation, we have proposed a multi-hypothesis initialization estimator. We have assessed the performance of the algorithm by means of computer simulations. Finally, we have confirmed the expected behavior of the algorithm through the processing of experimental data.

## Figures and Tables

**Table 1:** Parameters of the Calibrated Sensors Model.

Error	Accelerometer	Magnetometer
Noise	0.5 m/s/√h	0.2 mGauss/√Hz
Bias	10 mg	0.001 Gauss
Misalignment	2 mrad	2 mrad
Linear Scale Factor	0.17%	0.2%

## Data Availability

Not applicable.

## References

[1] Michel T., Genevès P., Fourati H., Layaïda N. On Attitude Estimation with Smartphones. Proceedings of the IEEE International Conference on Pervasive Computing and Communications.

[2] Harman R., Thienel J., Oshman Y. Gyroless attitude and rate estimation algorithms for the fuse spacecraft. Proceedings of the Flight Mechanics Symposium, NASA Goddard Space Flight Center.

[3] Santoni F., Bolotti F. Attitude determination of small spinning spacecraft using three-axis magnetometer and solar panel data. Proceedings of the IEEE Aerospace Conference (Cat. No.00TH8484).

[4] Zipfel P.H. (2007). Modeling and Simulation of Aerospace Vehicle Dynamics.

[5] Habibi S., Cooper S.J., Stauffer J.M., Dutoit B. (2008). Gun Hard Inertial Measurement Unit Based on MEMS Capacitive Accelerometer and Rate Sensor.

[6] Harking T.E. (2007). On the Viability of Magnetometer-Based Projectile Orientation Measurements.

[7] Changey S., Pecheur E., Bernard L., Sommer E., Wey P., Berner C. (2012). Real-Time Estimation of Projectile Roll Angle Using Magnetometers: In-flight Experimental Validation.

[8] Rogers J., Costello M., Harkins T., Hamaoui M. (2011). Effective Use of Magnetometer Feedback for Smart Projectile Applications. J. Inst. Navig..

[9] Renaudin V., Afzal M.H., Lachapelle G. (2010). Complete Triaxis Magnetometer Calibration in the Magnetic Domain. J. Sensors..

[10] Alonso R., Shuster M.D. (2002). Complete linear attitude-independent magnetometer calibration. J. Astronaut. Sci..

[11] Gebre-Egziabher D., Elkaim G.H., Powell J.D., Parkinson B.W. (2006). Calibration of Strapdown Magnetometers in the Magnetic Field Domain. J. Aerosp. Eng..

[12] Vasconcelos J.F., Elkaim G., Silvestre C., Oliveira P., Cardeira B. (2011). Geometric Approach to Strapdown Magnetometer Calibration in Sensor Frame. IEEE TAES.

[13] Foster C.C., Elkaim G.H. (2008). Extension of a two-step calibration methodology to include non-orthogonal sensor axes. IEEE TAES.

[14] Liu Y.X., Li X.S., Zhang X.J., Feng Y.B. (2014). Novel Calibration Algorithm for a Three-Axis Strapdown Magnetometer. Sensors.

[15] Wu Y., Luo S. (2016). On Misalignment between Magnetometer and Inertial Sensors. IEEE Sens. J..

[16] Combettes C., Changey S., Adam R., Pecheur E. (2018). Attitude and Velocity Estimation of a Projectile Using Low-Cost Magnetometers and Accelerometers.

[17] An L., Wang L., Liu N., Fu J., Zhong Y. (2019). A Novel Method for Estimating Pitch and Yaw of Rotating Projectiles Based on Dynamic Constraints. Sensors.

[18] Mok S.H., Byeon S.Y., Bang H., Choi Y. Attitude Dynamics Model-Based Gyroless Attitude Estimation for Agile Spacecraft. Proceedings of the 18th ICCAS.

[19] Deng Z. (2018). Roll Angle Measurement for a Spinning Vehicle Based on GPS Signals Received by a Single-Patch Antenna. Sensors.

[20] Psiaki M. (2001). Attitude sensing using a global-positioning-system antenna on a turntable. J. Guid. Control. Dyn..

[21] Teunissen P.G., Giorgi G., Buist P.J. (2011). Testing of a new single-frequency GNSS carrier phase attitude determination method: Land, ship and aircraft experiments. GPS Solut..

[22] Nusbaum U., Rusnak I., Klein I. (2020). Angular accelerometer-based inertial navigation system. J. Inst. Navig..

[23] Ohlmeyer E.J., Pepitone T.R. Guidance, Navigation and Control without Gyros: A Gun-Launched Munition Concept. Proceedings of the AIAA GNC Conference and Exhibit.

[24] Angrisano A. (2010). GNSS/INS Integration Methods. Ph.D. Thesis.

[25] The World Magnetic Model. https://www.ngdc.noaa.gov/geomag/WMM/DoDWMM.shtml.

[26] Gebre-Egziabher D., Elkaim G.H., Powell J.D., Parkinson B.W. (2000). A Gyro-Free Quaternion-Based Attitude Determination System Suitable for Implementation Using Low Cost Sensors.

[27] Wey P., Corriveau D., Saitz T., Ruijter W., Strömbäck P. BALCO 6/7-DoF Trajectory Model. Proceedings of the 29th International Symposium on Ballistics.

[28] Groves P.D. (2008). Principles of GNSS, Inertial, and Multisensor Integrated Navigation Systems.

[29] Xiong K., Liu L.D., Zhang H.Y. (2009). Modified Unscented Kalman Filtering and Its Application in Autonomous Satellite Navigation.

[30] Ohlmeyer E.J., Pepitone T., Miller B. Assessment of Integrated GPS/INS for the EX-171 Extended Range Guided Munition. Proceedings of the AIAA GNC Conference.

